# Comprehensive Analysis of lncRNA–mRNA Expression Profiles in Depression-like Responses of Mice Related to Polystyrene Nanoparticle Exposure

**DOI:** 10.3390/toxics11070600

**Published:** 2023-07-10

**Authors:** Qingping Liu, Wentao Hu, Yaling Zhang, Jie Ning, Yaxian Pang, Huaifang Hu, Meiyu Chen, Mengqi Wu, Mengruo Wang, Peihao Yang, Lei Bao, Yujie Niu, Rong Zhang

**Affiliations:** 1 Department of Toxicology, Hebei Medical University, Shijiazhuang 050017, China; lqp0830_academic@126.com (Q.L.); hwt1399634374@outlook.com (W.H.); zyl19940129@126.com (Y.Z.); jning0101@163.com (J.N.); pangyaxian@hebmu.edu.cn (Y.P.); hhf09260818@163.com (H.H.); charleschenmeiyu@163.com (M.C.); wmqacademic@163.com (M.W.); wangmengruo2023@163.com (M.W.); yphhbmu@163.com (P.Y.); 2 Occupational Health and Environmental Health, Hebei Medical University, Shijiazhuang 050017, China; baolei0719@126.com (L.B.); yjniu@hebmu.edu.cn (Y.N.); 3 Hebei Key Laboratory of Environment and Human Health, Hebei Medical University, Shijiazhuang 050017, China

**Keywords:** nano-polystyrene, depression-like responses, LncRNA, RNA-sequencing, ceRNA

## Abstract

Plastics in the environment can break down into nanoplastics (NPs), which pose a potential threat to public health. Studies have shown that the nervous system constitutes a significant target for nanoplastics. However, the potential mechanism behind nanoplastics’ neurotoxicity remains unknown. This study aimed to investigate the role of lncRNA in the depressive-like responses induced by exposure to 25 nm polystyrene nanoplastics (PS NPs). Forty mice were divided into four groups administered doses of 0, 10, 25, and 50 mg/kg via gavage for 6 months. After conducting behavioral tests, RNA sequencing was used to detect changes in mRNAs, miRNAs, and lncRNAs in the prefrontal cortex of the mice in the 0 and 50 mg/kg PS NPs groups. The results revealed that mice exposed to chronic PS NPs developed depressive-like responses in a dose-dependent manner. It was demonstrated that 987 mRNAs, 29 miRNAs, and 116 lncRNAs were significantly different between the two groups. Then, a competing endogenous RNA (ceRNA) network containing 6 lncRNAs, 18 miRNAs, and 750 mRNAs was constructed. Enrichment results suggested that PS NPs may contribute to the onset of depression-like responses through the activation of axon guidance, neurotrophin-signaling pathways, and dopaminergic synapses. This study provided evidence of the molecular relationship between PS NPs and depression-like responses.

## 1. Introduction

In recent years, the threat of plastic pollution to human health has become a public health issue of global concern [1,2]. Global plastic production has soared over the past few decades due to the product’s widespread use in our lives and industries. It has been reported that the global production of plastics rose from 1.5 million tons in the 1950s to 359 million tons in 2018 [3]. At the same time, a large amount of plastic waste has been sent to landfills, resulting in the pollution of the environment. In 2016, an estimated 19 to 23 million tons of plastic waste generated globally reached water systems, and annual emissions could reach 53 million tons yearly by 2030. The current amount of plastic production is about 450 million tons annually, which is projected to double by 2045 [4,5]. However, most plastics are not easily degradable, and the continuous decomposition of plastic products as a result of thermal, chemical, and biological processes leads to the creation of microplastics (MPs) [6]. MPs can be further broken down into smaller-particle-size nanoparticles (NPs < 0.1 μm) [7]. Studies have shown that NPs may enter the body via multiple pathways and can be distributed to different tissues through the circulatory system [8,9,10]. At present, polystyrene (PS) is one of the most-produced plastic materials [11]. Previous studies have evidenced that PS NPs can cross the blood–brain barrier into the brain [12,13], leading to various neurological disorders, such as anxiety [14] and neurobehavioral impairments [15,16].

Depression is one of the most common mental illnesses [17]. It was reported that mental illness would be the leading cause of global health-related burdens by 2020, and depression is one of the major contributors to this burden [18]. The World Health Organization (WHO) reported that depression ranked as the third leading contributor to the global burden of disease in 2004, and it is expected to rise to first place by 2030 [19]. Previous studies have shown that environmental factors, including nanoparticle exposure, contribute to the onset of depressive disorders [20,21,22]. It has been shown that the deposition of nanomaterials is associated with increased NO levels, increased thiobarbituric acid reactive species, and lower acetylcholinesterase activity in the brain (leading to cognitive impairment) [23]. Our previous research has shown that chronic PS exposure can induce cognitive impairment in mice through damage to synaptic functions [24]. However, the neurotoxic mechanisms of PS NPs still deserve to be fully investigated.

Researchers have demonstrated that long noncoding RNAs (lncRNAs) are highly expressed in the brain and play a critical role in neural stem cell maintenance, brain patterns, synaptic and stress responses, and neuroplasticity [25]. Evidence has suggested that lncRNA deregulation is one of the potential mechanisms behind many neurological disorders, including depression [26,27]. Previous study demonstrated that lncRNA has shown potential value as a diagnostic and therapeutic biomarker for depression [27]. LncRNAs can regulate mRNA stability and translation in the cis or trans formation and interact with miRNAs to regulate mRNA transcription [28]. Furthermore, a previous study demonstrated that PS NPs (1 μg/L) increased the expression of linc-2, linc-9, linc-18, and linc-61 and decreased the expression of linc-50 [29]. RNA-sequencing (RNA-seq) results from another study showed differential expression of circRNA and lncRNA in the lung tissue of rats exposed to PS MPs [30]. However, the mechanism of lncRNAs in the neurotoxicity induced by PS NPs exposure still requires exploration.

In the present study, C57BL/6 mice were treated with PS NPs via gavage for 6 months. Our findings suggest that chronic exposure to PS NPs can lead to injury in the prefrontal cortex (PFC), leading to depressive-like responses in mice. RNA-seq was used to detect differentially expressed transcripts in the PFC to elucidate the mechanics behind the depression-like responses caused by PS NPs. The potential mechanism by which PS NPs cause depression-like responses in mice is as follows: the dysregulation of lncRNAs activates multiple important pathways, including axon guidance, dopaminergic synapses, and neurotrophin signaling pathways. The present study provides potential strategies for the early diagnosis of depression-like responses induced by PS NPs and deeply explains the potential pathological mechanisms of PS-NP-induced depressive behavior in mice.

## 2. Materials and Methods

### 2.1. Characterization of PS NPs

We purchased 25 nm polystyrene samples from Bangs Laboratories, Inc. (Fishers, IN, USA). PS NPs were characterized via scanning electron microscopy (SEM) using an IT-500HR instrument (JEOL, Tokyo, Japan). Size distribution and zeta potential of PS NPs were examined using dynamic light scattering (DLS) at 25 °C (PALS, Brookhaven National Laboratory, New York, NY, USA).

### 2.2. Animals and Treatment

Five-week-old male C57BL/6 mice were purchased from Vital River Laboratory (Beijing Vital River Laboratory Animal Technology Co., Ltd., Beijing, China) and housed in individually ventilated cages. The mice were maintained in a temperature- (25 ± 3 °C) and humidity (50 ± 5%)-controlled environment under a 12 h light/dark cycle. Water and standard laboratory feed were made available ad libitum.

**Figure 1:**
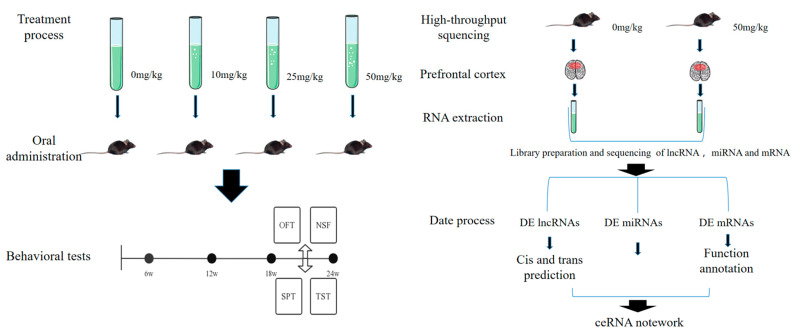
Flow chart illustrating the design of the experiment.

A total of 40 mice were divided into a control group, a low-dose group (10 mg/kg body weight (bw) PS NPs), a middle-dose group (25 mg/kg bw PS NPs), and a high-dose group (50 mg/kg bw PS NPs). PS NPs were dissolved in ultrapure water and sonicated for 5 min before each application. Mice in PS NPs groups were administered different concentrations of PS NPs via oral gavage daily for 6 months, while the control group was given equal amounts of distilled water. After the behavioral tests, mice were euthanized via cervical dislocation under anesthesia (1% sodium pentobarbital intraperitoneal injection), and the prefrontal cortex of each mouse was removed rapidly on ice and stored at −80 °C. The layout of our study is shown in Figure 1. The Animal Care and Use Committee of Hebei Medical University (Hebei, China) approved the experimental procedures.

### 2.3. Behavioral Tests

#### 2.3.1. Open Field Test (OFT)

The states of nervousness and anxiety in the mice were assessed using OFT and with reference to a previous study [31]. The experiments were conducted in a 30 cm × 30 cm × 25 cm square white square box. Mice were free to move around, and each mouse was observed for 5 min. The time and distance travelled in an open field were recorded and analyzed with a digital tracking system.

#### 2.3.2. Novelty-Suppressed Feeding Test (NSF)

The NSF assay is commonly used to evaluate depressive-like states in mice. Mice were subjected to a 24 h water-and-food fast before the novel inhibition of feeding experiment. On the following day, each mouse was subjected to a test individually in a corner of the field, and the latency period regarding the point at which each mouse started feeding was recorded. Observations were made for a maximum of 10 min.

#### 2.3.3. Sucrose Preference Test (SPT)

The SPT was used to detect anhedonia and evaluate depressive behavior in animals. Every mouse was housed in a cage with two bottles, containing a solution of 3% sucrose and water, respectively. Mice were allowed to freely choose to drink from either of the two water bottles. Liquid intake was measured daily for 3 days, and bottles were inverted daily to prevent position preference bias. The sucrose preferences of the mice in the different treatment groups were calculated as the sucrose solution intake as a percentage of the total intake.

#### 2.3.4. Mouse Tail Suspension Test (TST)

Depressive-like behavior was assessed through the tail suspension test, which was performed according to a previous publication [32]. The mice were suspended 50 cm above the floor with adhesive tape, which was wrapped around 1 cm from the tip of the tail. Mice were videotaped for 6 min, and the total immobility time was recorded (defined as the time taken for the limbs to become immobile). During the test, mice were separated from each other to prevent possible visual and acoustic interplay.

### 2.4. RNA Sequencing

Total RNA of PFC tissues in 0 and 50 mg/kg PS NP groups was extracted using Trizol reagent (Invitrogen, Carlsbad, CA, USA). The quality and integrity of total RNA were evaluated using NanoDrop ND-1000 (Wilmington, CA, USA). RNA integrity numbers (RIN) >7.0 were used for subsequent analysis. 

Ribosomal RNA was removed from the total RNA using the Ribo-Zero™ rRNA Removal Kit (Illumina, San Diego, CA, USA). High-temperature treatment was conducted to cleave the RNA into small fragments, and the cleaved RNA fragments were reverse-transcribed to create a cDNA library. Libraries were then sequenced using an Agilent 2100 Bioanalyzer and accurately quantified using PCR. The sequencing libraries were generated and sequenced by LC Biotech (Hangzhou, China). Ultimately, we used StringTie and Edger to assess the expression levels of transcripts in the PFC of the brain. We excluded transcripts that were identical to known mRNAs or less than 200 bp in length. Then, we used Coding Potential Calculator (CPC) [33] and Coding-Non-Coding Index (CNCI) [34] to predict the coding ability of individual transcripts. Transcripts with a CPC score ≥ −1 and a CNCI score ≥ 0 were considered lncRNAs. 

A total of 1 µg of total RNA was used to prepare miRNA libraries. We performed single-end sequencing (1 × 50 bp) according to the standard protocol. The raw reads were processed using software to remove extraneous material, low-complexity reads, and duplicates. Next, we mapped sequences with a length of 18–26 nt to mouse miRNAs in miRBase 22.0. Sequences matching the mature miRNA hairpins of the mice were considered known miRNAs, while sequences matching the other arm of known mouse precursor hairpins were considered novel 5p or 3p-derived miRNAs. The other sequences were linked with other species precursors in miRBase 22.0, and the matched pre-miRNAs were compared with the mouse genome to determine their genomic locations. 

### 2.5. Target Gene Prediction of lncRNAs

The cis–trans regulation of lncRNAs is an important process determining the way in which lncRNAs function, and we used a Python script to select the coded portion of the 100,000 upstream and downstream ranges. A |log_2_fold change (FC)| > 1, with *p* < 0.05, was considered a meaningful cis-prediction; for the trans-prediction, we selected a Pearson correlation coefficient greater than 0.99.

### 2.6. Construction of the Competing Endogenous RNA (ceRNA) Regulatory Network

To clarify the potential biological functions of the differentially expressed functions, we selected the top 5 up- and top 5 down-regulated functions to build a ceRNA network (Table S1). To identify the potential combinations, we used TargetScan and miRanda to make predictions. The thresholds applied were a TargetScan score ≥ 50 and a miRanda energy value < −10, respectively. The ceRNA network was completed using Cytoscape (Version 3.7.1; https://cytoscape.org/; accessed on 1 May 2023). 

### 2.7. Construction of the Protein–Protein Interaction (PPI) Network

Search Tool for the Retrieval of Interacting Genes/Proteins (STRING) was used to develop the interaction model. We applied STRING (version 10.5; https://string-db.org/; accessed on 1 May 2023) with a selected confidence level of >0.4 to determine the potential relationships between differentially expressed mRNAs. Then, the PPI network was completed using Cytoscape. 

### 2.8. Bioinformatic Analyses

Gene Ontology (GO) term enrichment analysis was performed to obtain annotation and enrichment information. Biological processes (BPs) for GO enrichment analysis were analyzed using the BINGO plugin of Cytoscape. *p* < 0.05 was considered statistically significant for annotation compared to control. The Kyoto Encyclopedia of Genes and Genomes (KEGG) functional annotation and pathway enrichment analysis of differentially altered mRNAs were performed using Kobas 3.0 [35]. *p* < 0.05 was used as the threshold, and those pathways meeting this condition were defined as significantly enriched pathways in the transcriptome. Visualization through ChiPlot online website (https://www.chiplot.online/; accessed on 1 May 2023).

### 2.9. Real-Time Quantitative PCR (qRT-PCR) Validation

The differentially expressed lncRNAs, miRNAs, and mRNAs were randomly selected for qRT-PCR analysis. Total RNAs were extracted from the PFC of the brain using Trizol (Invitrogen, Carlsbad, CA, USA). cDNA synthesis of miRNA was performed using miRNA 1st Strand cDNA Synthesis Kit (via stem-loop) (vazyme, Nanjing, China), and cDNA synthesis of lncRNA and mRNA was performed using RevertAid First Strand cDNA Synthesis Kit (Thermoscientific, USA). The experimental primers (Table S2) were designed and synthesized by Sangon Biotech (Shanghai, China).

### 2.10. Statistical Analysis

SPSS 20.0 (IBM, SPSS, Chicago, IL, USA) was used for statistical analysis, and data are expressed as mean ± standard deviation. The results from the GO and KEGG pathway analyses were processed using Fisher’s Exact test on the website. Multiple comparisons between all groups of the behavioral experiment were performed using one-way analysis of variance (ANOVA). *p* < 0.05 was considered a statistically significant difference.

## 3. Results

### 3.1. Characterization of PS NPs

In previous studies conducted by our group, all the analyzed PS NPs were nearly spherical and well dispersed [24]. SEM revealed nanoparticle sizes between 20 and 30 nm. The size of the NPs was 56.32 nm ± 1.42 nm in an aqueous fluid at 25 °C. The measured zeta potential of these PS NPs was −41.90 mV.

### 3.2. PS NPs Exposure Induced Depression-like Responses in Mice

**Figure 2:**
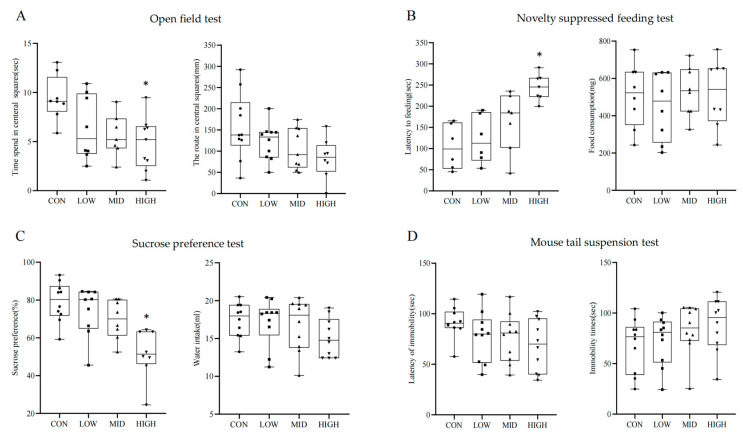
Changes in behavior after PS NP exposure in mice. (**A**) Time spent and route taken in central squares in open field test; (**B**) latency of feeding in novelty-suppressed feeding and food consumption test; (**C**) sucrose preference and total water intake; (**D**) the immobility times and immobility latency in mouse tail suspension test. The data are presented as mean ± SD. *n* = 8. * *p* < 0.05 vs. CON.

Compared to the 0 mg/kg group, the time spent in central squares was significantly decreased in the 50 mg/kg group (*p* < 0.05). Compared to the control group, the length of the route travelled in the central squares in the low-dose, mid-dose, and high-dose groups was slightly decreased (*p* > 0.05). The results of OFT are presented in Figure 2A, which indicates that PS NP exposure induced a decrease in autonomous activity.

In Figure 2B, the NSF results show that compared to the control group, the latency of feeding was significantly increased in the 50 mg/kg group (*p* < 0.05), but there was no significant difference in the amount of food consumed (*p* > 0.05).

The SPT results showed that the preference for sucrose in the 50 mg/kg group was significantly decreased compared with that of the control group in Figure 2C (*p* < 0.05). The differences in the amounts of water consumed were not statistically significant in the different groups (*p* > 0.05).

The TST results are shown in Figure 2D. Compared to the control group, all groups showed a slight decrease in the latency of immobility and a slight increase in immobility time (*p* > 0.05). All the above behavioral tests suggested that PS NPs induced depressive-like behaviors in the mice.

### 3.3. LncRNA, miRNA, and mRNA Expression Profiles

**Figure 3:**
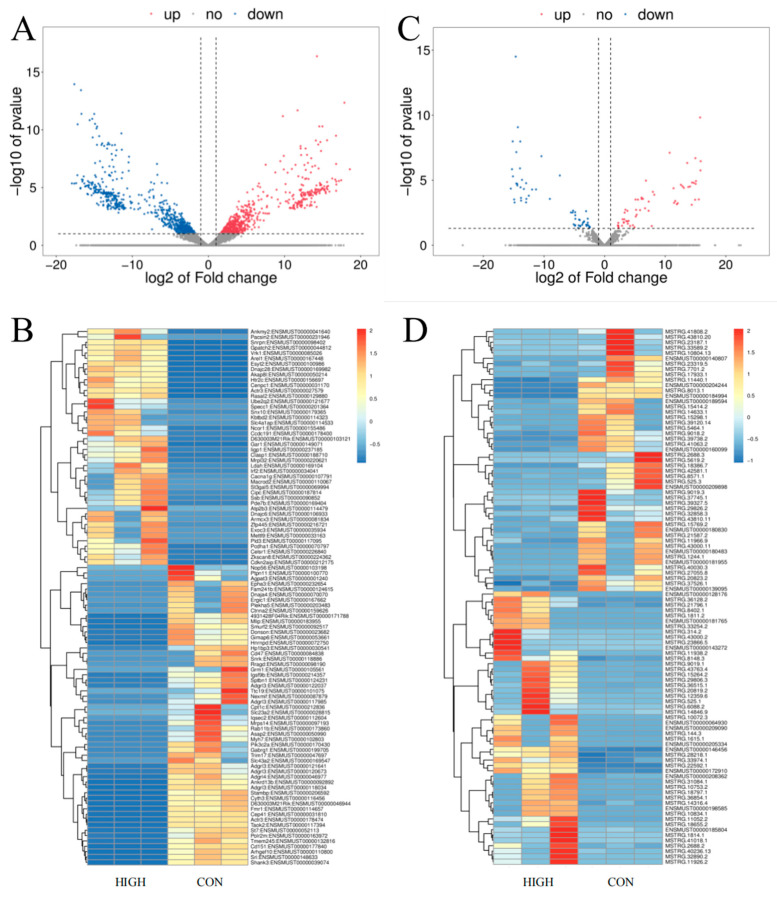
Volcano plots and heatmap of DE mRNAs and DE lncRNAs in PFC tissues following exposure to 50 mg/kg of PS NPs compared with the control mice. (**A**) Volcano plots of mRNAs; (**B**) heatmap of mRNAs; (**C**) volcano plots of lncRNAs; (**D**) heatmap of lncRNAs. Blue points represent down-regulated RNAs, while red points represent up-regulated RNAs. Warm shades indicate increased expression, whereas cool shades indicate reduced expression. *n* = 3. PFC: prefrontal cortex. HIGH: 50 mg/kg PS-NP exposed group; CON: control group.

The different mRNAs (DE mRNAs) and lncRNAs (DE lncRNAs) expressed in the 50 mg/kg group were shown using a Volcano plot and a heatmap (*p* < 0.05 and |log_2_FC| > 1.0) (Figure 3). In total, there were 987 DE mRNAs (477 up–regulated and 510 down–regulated) (Figure 3A,B) and 116 DE lncRNAs (59 up–regulated and 57 down–regulated) (Figure 3C,D). The specific details of the top five up–regulated and down–regulated mRNAs are listed in Table S3. The most up–regulated mRNA was ENSMUST00000106933 (log_2_FC: 18.58), and the most down–regulated mRNA was ENSMUST00000152117 (log_2_FC: −17.94). Among the top five up–regulated and top five down–regulated DE lncRNAs, MSTRG.11938.2 (log_2_FC: 15.85, *p* < 0.05) and MSTRG.41808.2 (log_2_FC: −15.47, *p* < 0.05) were the most up–regulated or down–regulated lncRNAs. 

The miRNA-sequencing results showed that there were 29 differentially expressed miRNAs (DE miRNAs) (17 upregulated and 12 downregulated) (*p* < 0.05). Among them, seven miRNAs (miR-667-3p, miR-3084-3p, miR-3572-3p, PC-3p-25230, PC-3p-12229, PC-3p-33965, and miR-540-3p) were not homologous with human miRNAs (Table S4).

### 3.4. Function and Pathway Analysis of the Identified DE mRNAs and DE lncRNAs

**Figure 4:**
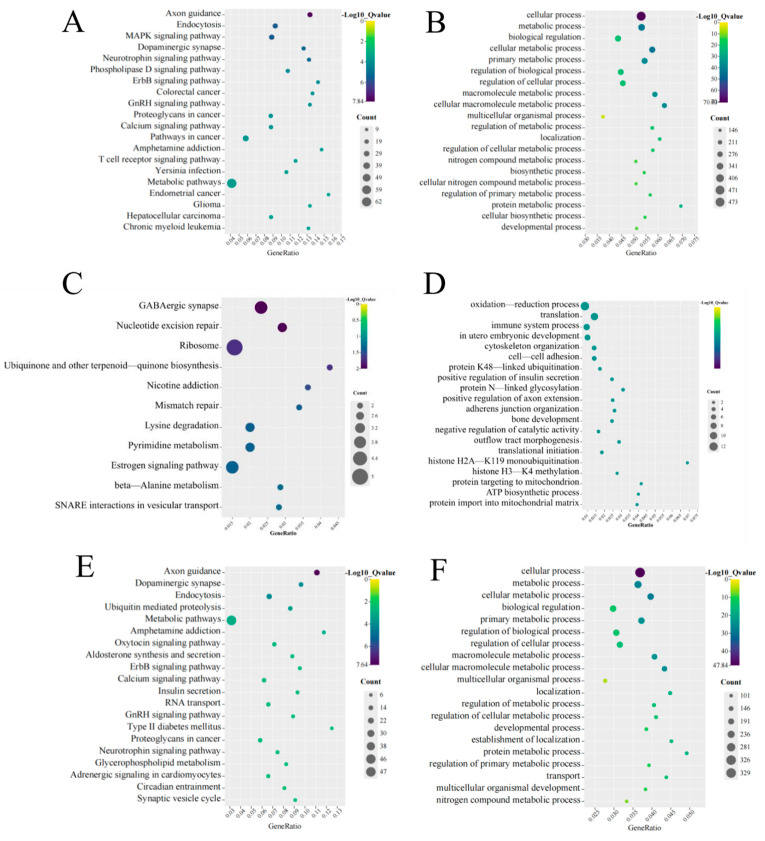
Enrichment of functions and signaling pathways of mRNAs. (**A**) The top 20 KEGG pathways in DE mRNAs; (**B**) the most enriched GO term of DE mRNAs; (**C**) the top 20 KEGG pathways of DE lncRNA cis–target genes; (**D**) the most enriched GO term of DE lncRNA cis–target genes; (**E**) the top 20 KEGG pathways of DE lncRNA trans–target genes; (**F**) the most enriched GO term of DE lncRNA trans–target genes.

To ascertain the function of DE mRNAs, we performed KEGG and GO analyses. KEGG analysis revealed that axon guidance, endocytosis, the MAPK signaling pathway, dopaminergic synapses, and the neurotrophin signaling pathway were the top five pathways. The significant pathways as determined via KEGG enrichment analysis are shown in Figure 4A. GO analysis showed that the first five enriched BP function terms for the DE mRNAs were cellular processes, metabolic processes, biological regulation, cellular metabolic processes, and primary metabolic processes (Figure 4B).

We explored the potential functions of DE lncRNAs. KEGG analysis was performed on cis regulation target genes; we found that GABAergic synapse, nucleotide excision repair, ribosome, ubiquinone, and other terpenoid-quinone biosynthesis and nicotine addiction pathways were the top five pathways (Figure 4C). Oxidation–reduction processes, translation, immune system processes, in utero embryonic development, and cytoskeletal organization were the top five BP functions according to GO analysis of the target genes (Figure 4D). 

For the trans regulation target genes, we found that axon guidance, dopaminergic synapse, endocytosis, ubiquitin-mediated proteolysis, and metabolic pathways were the top five pathways (Figure 4E). The top five BP functions in terms of GO enrichment for DE mRNAs were cellular processes, metabolic processes, cellular metabolic processes, biological regulation, and primary metabolic processes (Figure 4F).

### 3.5. Construction of the LncRNA–miRNA-mRNA ceRNA Regulatory Network

To explore how DE LncRNAs regulate downstream roles, we constructed an LncRNA–miRNA–mRNA ceRNA network. We selected 10 lncRNAs, including the top 5 up– and top 5 down–regulated lncRNAs, to construct a ceRNA network using a bioinformatics approach. In the end, 199 lncRNA–miRNA–mRNA interactions were obtained, including 3 up– and 3 down–regulated lncRNAs, 12 up– and 6 down–regulated miRNAs, and 334 up– and 416 down–regulated mRNAs.

### 3.6. Function Analysis of mRNAs in ceRNA Network

**Figure 5:**
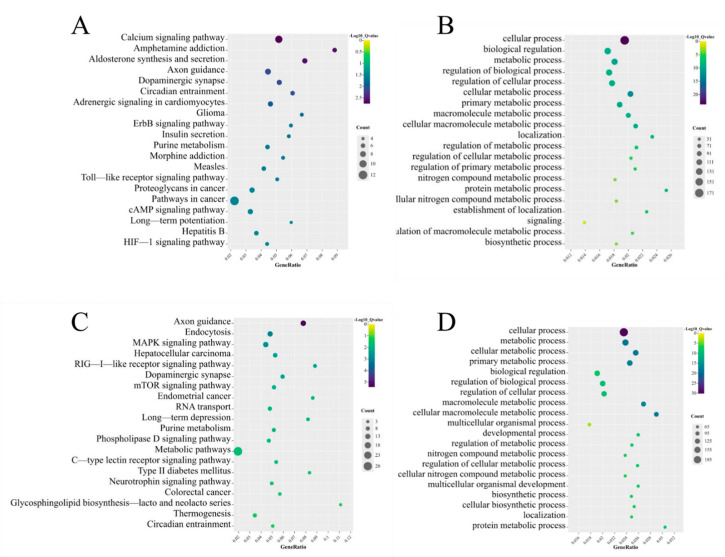
Functional enrichment analysis of mRNAs determined using ceRNA network. (**A**) The top 20 KEGG pathways of target genes according to up–regulated lncRNAs; (**B**) the most enriched GO term of target genes according to up–regulated lncRNAs; (**C**) the top 20 KEGG pathways of target genes according to down–regulated lncRNAs; (**D**) the most enriched GO term of target genes according to down–regulated lncRNAs.

To investigate the possible pathways involving mRNAs in the ceRNA network, we performed KEGG and GO analyses on mRNAs targeted by up– and down–regulated lncRNAs. The up–regulated target genes determined using KEGG enrichment analysis for the top 20 pathways are shown in Figure 5A. We found that the calcium signaling pathway, amphetamine addiction, aldosterone synthesis and secretion, axon guidance, and the dopaminergic synapse pathway were the top five pathways. In the GO enrichment analysis, the top five enriched BP functional terms for GO enrichment analysis were cellular processes, biological regulation, metabolic processes, regulation of biological processes, and regulation of cellular processes (Figure 5B).

For the down–regulated lncRNAs, KEGG analysis showed that axon guidance, endocytosis, the MAPK signaling pathway, hepatocellular carcinoma, and RIG-I-like receptor were the top five pathways (Figure 5C). Cellular processes, metabolic processes, cellular metabolic processes, biological regulation, and primary metabolic processes were their main BP functional terms (Figure 5D).

### 3.7. PPI Network Analysis of mRNAs

**Figure 6:**
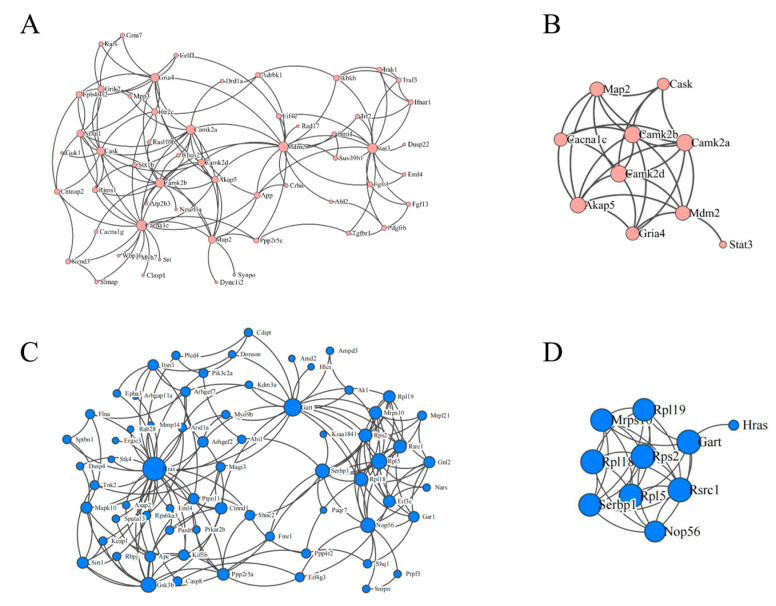
Protein–protein interaction (PPI) network of differentially expressed mRNAs. (**A**) The up-regulated genes in the cerna network; (**B**) the top ten genes screened using cytohubba; (**C**) the down-regulated genes in the cerna network; (**D**) the ten genes screened using cytohubba. The size of each point represents the level of importance in the network.

**Figure 7:**
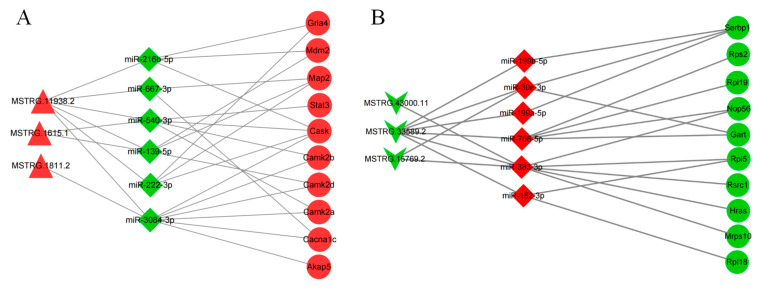
The ceRNA network concerning the selected lncRNAs. (**A**) Construction of the ceRNA regulatory network (miRNA down-regulation); (**B**) construction of the ceRNA regulatory network (miRNA up-regulation). In the graph, red indicates upward adjustment, while green indicates downward adjustment.

We constructed a PPI network of the mRNAs in the ceRNA network according to up– and down–regulation relationships (Figure 6A,C). Analysis was performed using the Cytohabba plugin of the Cytoscape software. The top ten important targets of up- and down-regulation were screened using this plugin (Figure 6B,D). We established two ceRNA sub-networks containing 3 lncRNAs, 6 miRNAs, and 10 mRNAs from the screened target genes (Figure 7A,B). These subnetworks may reveal regulatory pathways of important mRNAs.

For instance, we predicted that Grias could be regulated by two miRNAs, namely, miR-216b-5p and miR-222-3p. MSTRG.11938.2 can regulate Grias expression through competitive binding to miR-216b-5p. Cask can be regulated by miR-3084-3p, miR-540-3p, miR-216b-5p, and miR-222-3p. MSTRG.1615.1 regulates Cask expression through competitive binding to miR-216b-5p.

### 3.8. qRT-PCR Experiments

**Figure 8:**
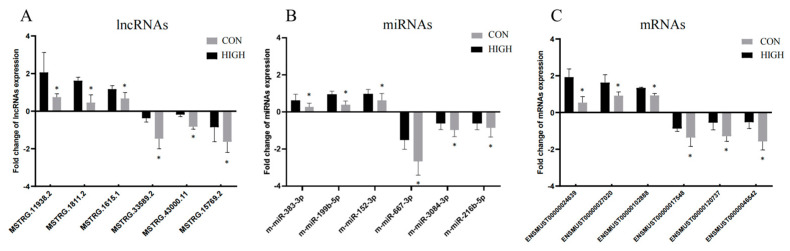
Validation of lncRNA, miRNA, and mRNA expression. (**A**) The expression levels of six lncRNAs in the PFC were validated using qRT-PCR and RNA-seq analyses; (**B**) the six miRNAs that were validated; (**C**) the six mRNAs that were validated. The Y axis indicates the log_2_ (fold change) in the lncRNAs and mRNAs and the fold change in the miRNAs. The negative values correspond to down-regulated lncRNAs, miRNAs, and mRNAs, whereas the positive values correspond to up-regulated lncRNAs, miRNAs, and mRNAs. The data are presented as mean ± SD. *n* = 6. PFC: prefrontal cortex. HIGH: 50 mg/kg PS NP group; CON: control group. * *p* < 0.05 vs. CON.

We used qRT-PCR experiments to analyze six lncRNAs, six miRNAs, and six mRNAs selected from the ceRNA network. The expression of the selected RNAs was consistent with the sequencing results (Figure 8). 

## 4. Discussion

In this study, we administered PS NPs to mice via gavage for 6 months and explored their behavioral neurotoxicity and potential mechanisms. The results revealed that the mice in the 50 mg/kg group showed depression-like responses. Furthermore, we used high-throughput sequencing to detect the expression levels of transcripts that regulated the expression of related mRNAs. In addition, it was found that mRNAs related to axon guidance, the MAPK signaling pathway, and dopaminergic synaptic pathways functioned abnormally after PS NP exposure. Finally, our study suggested that the cis and trans regulation of lncRNAs and ceRNA regulation were potential mechanisms of neurotoxicity induced by PS NPs.

Studies have shown that humans may be exposed to 0.1–5 g of microplastics per week [36]. Based on this populational exposure and interspecies dose conversion, 10, 25, and 50 mg/kg concentrations of polystyrene nanoparticles were used in the present study [37]. The exposure dose for mice was calculated to be 50 mg/kg, which is equivalent to a human intake of 2.7 g of PS NPs per week. In this study, behavioral tests showed a significant decrease in spatial cognition and exploratory behavior, which indicated a depression-like response in the mice in the 50 mg/kg group. In another study on PS NP toxicity, cognitive deficits (determined based on object recognition tests) occurred in mice after exposure to PS NPs for 3 days via the intraperitoneal route (14.6 ng/kg), but there was no change in locomotion [25]. The conflicting nature of these results might have been caused by the duration and concentration.

Based on our RNA-seq results, we identified 987 differentially expressed mRNAs. KEGG analysis of DE mRNAs revealed that axon guidance, the MAPK signaling pathway, the dopaminergic synapse pathway, and the neurotrophin signaling pathway were activated in the PS-NP-exposed mice. Our results suggested that abnormal neurogenesis in mice exposed to PS NPs may be responsible for depressive symptoms. These activated pathways are closely linked to neurogenesis, and overlapping genes in these pathways may play an important role. A previous study showed that Ptpn11 played an important role in excitatory synapses [38]. In another study, calcium/calmodulin-dependent protein kinase II Alpha (Camk2a) played a significant role in the development of depression [39]. The axon guidance, dopaminergic synapse, and Erb-b2 receptor tyrosine kinase 2 (ErbB) signaling pathways were shown to be the pathways it may affect. Some other DE mRNAs controlled by these pathways, such as glycogen synthase kinase-3 beta (Gsk3b) and the protein phosphatase 3 catalytic subunit alpha isoform (Ppp3ca), have also been suggested to be potential contributors to the development of depression. It has been shown that the deletion of neuronal Gsk3b decreases the stability of dendritic spines and weakens excitatory synaptic transmission [40]. In one study, Ppp3ca was highly expressed in neurons and involved in mitochondrial biogenesis [41,42]. 

Previous studies have suggested that changes in miRNA expression may influence the development of depression [43,44]. In our study, we identified 29 DE miRNAs. MiR-98-5p plays a vital role in the antidepressant effect of ketamine, which could be a potential target for antidepressant treatment [45]. Evidence has suggested that elevated miR-199a-5p levels could regulate depression by targeting Wnt family member 2 (Wnt2) signaling to brain-derived neurotrophic factor (BDNF) [46]. Our study showed that miR-98-5p and miR-199a-5p were significantly altered in the 50 mg/kg group, which could partly explain the depressive symptoms of the mice after exposure.

There are various mechanisms for the lncRNA regulation of target genes, and the most common forms of regulation are cis and trans regulation [47]. Based on the results of RNA-seq, we predicted the target genes for the differentially expressed lncRNAs in cis and trans regulation. A total of 16 mRNAs were obtained for cis-regulation, and 621 mRNAs were obtained for trans-regulation. The KEGG results suggested that lncRNAs regulated target genes related to axon guidance, dopaminergic synapses, endocytosis, ubiquitin-mediated proteolysis, metabolic pathways, etc., which affect the development of depression. The Syntaxin 1B (Stx1b) gene was involved in the activation of these pathways. Studies have shown that Stx1b is an essential component of the presynaptic neurotransmitter release mechanism and is essential for neurotransmission [48]. In our study, Stx1b might have been altered by MSTRG.36854.1 cis regulation. Another important regulatory pathway of lncRNA is trans regulation; we also predicted the trans regulation of lncRNAs in the sequencing results. Trans-regulatory KEGG analysis revealed that pathways associated with neurodevelopment, such as “Axon guidance”, “dopaminergic synapses”, and “ubiquitin-mediated protein hydrolysis”, were essential pathways for the enrichment of the target genes. Genes that overlap under the pathway include H-ras proto-oncogene (Hras) and Ppp3ca. Hras may be involved in the development of depression via affecting these pathways. Previous studies demonstrated that the destabilization of Hras led to abnormal neuronal development [49], and Hras was shown to be one of the metabolite targets of potential drug proteins for the treatment of depression [50]. Hras was predicted to be a trans-regulatory target gene for ENSMUST00000181955, ENSMUST00000180483, and MSTRG.43000.11.

LncRNAs can regulate the binding of endogenous miRNAs to their target genes through the sponge-effect-based adsorption of specific miRNAs or the competitive binding of miRNAs to achieve the regulation of their target gene functions [28]. There is growing evidence that lncRNAs can influence disease processes through the ceRNA pathway, and their abnormal changes may lead to many kinds of diseases, including neurological diseases and neurodegenerative disorders [51]. In our study, we selected the top five up- and down-regulated lncRNAs to construct the ceRNA network. We constructed an up-regulation network with 3 lncRNAs, 6 miRNAs, and 321 mRNAs and a down-regulation network with 3 lncRNAs, 12 miRNAs, and 398 mRNAs. The KEGG results showed that the calcium signaling pathway, axon guidance, and dopaminergic synapses were significantly activated. The overlapping genes in these activation pathways were Camk2a and calcium/calmodulin-dependent protein kinase II delta (Camk2d), which have been linked to the development of depression [52]. Using ceRNA analysis, we obtained eight axes regulating Camk2a and Camk2d. Among these, MSTRG.11938.2 was encoded by the parental gene Neuregulin 3 (Nrg3). Nrg3 has been demonstrated to affect dendritic and axonal growth, which may be broadly involved in the development of neurodegenerative diseases [53,54,55]. In our demonstration of the up-regulated ceRNA network, MSTRG.11938.2 adsorbed miR-3084-3p through the sponge effect and subsequently regulated the mRNA expression of Camk2a and Camk2d, thus affecting axon guidance and the calcium signaling pathway. We computed the up-regulation network using the Cytohubba plugin in Cytoscape and found that both Camk2a and Camk2d were on major nodes of the up-regulated ceRNA network mRNA, suggesting that Camk2a and Camk2d play an active role in depression [56]. This result was consistent with the enrichment of the typical pathways above and further indicated that MSTRG.11938.2, MSTRG.1615.1, and MSTRG.1811.2 regulate Camk2a through a ceRNA mechanism. In the down-regulated ceRNA network, KEGG analysis showed that axon guidance, the MAPK signaling pathway, and the mTOR signaling pathway were significantly activated. Hras was one of the important overlapping genes. By predicting the ceRNA network, we obtained three regulatory networks concerning the gene. MSTRG.15769.2, MSTRG.43000.11, and MSTRG.33589.2 may regulate the expression of HRAS through miR-383-3p. 

## 5. Conclusions

This study shows that chronic exposure to PS NPs led to depression-like responses in mice. Sequencing analysis provided a differential expression profile of lncRNAs, miRNAs, and mRNAs, which are useful for understanding the molecular mechanisms of neurotoxicity induced by PS NPs. The ceRNA network of MSTRG.11938.2 functioned via the sponge adsorption of miR-139-5p, miR-216b-5p, miR-222-3p, miR-3084-3p, miR-540-3p, and miR-667-3p and regulated Akap5, Cacna1c, Camk2a. Camk2b, Camk2d, Cask, Gria4, Map2, and Mdm2; MSTRG.15769.2, MSTRG.43000.11, and MSTRG.33589.2 sponged mir-383-3p to regulate HRAS. Our findings provide evidence that provides insight into the depression-like responses induced by PS NPs, suggesting that PS NPs potentially induce the production of depression-like responses through the activation of axon guidance, the neurotrophin signaling pathway, and dopaminergic synapse-related lncRNAs.

## Data Availability

Data and methods used in the research are presented in sufficient detail in the manuscript.

## Supplementary Materials

The following supporting information can be downloaded at: https://www.mdpi.com/article/10.3390/toxics11070600/s1. Table S1. The detailed information of the top five up-regulated and down-regulated lncRNAs. Table S2. The detailed information of primer sequences. Table S3. The detailed information of the top five up-regulated and down-regulated mRNAs. Table S4. The detailed information of differentially expressed miRNAs
