# Supplementary material for: Comprehensive Analysis of lncRNA–mRNA Expression Profiles in Depression-like Responses of Mice Related to Polystyrene Nanoparticle Exposure
Source: Toxics. 2023 Jul 10;11(7):600. doi: 10.3390/toxics11070600 (PMC10386552; doi:10.3390/toxics11070600)
Supplement: Supplementary file 1 [file toxics-11-00600-s001.zip › toxics-2454504-supplementary.pdf]

Supplementary Materials

Table S1. The detailed information of the top five up-regulated and down-regulated lncRNAs.

Transcript ID	length	log_2FC	regulation	*P*-value	FDR	chromosome
MSTRG.11938.2	30597	15.85	up	3.44E-07	0.000148291	chr14
MSTRG.1811.2	7160	15.81	up	1.75E-06	0.000577	chr1
MSTRG.36128.2	2939	15.75	up	1.50E-10	2.94577E-07	chr7
MSTRG.31084.1	244	15.53	up	0.000299043	0.022645844	chr5
MSTRG.1615.1	2805	15.07	up	1.15E-05	0.002319591	chr1
MSTRG.15298.1	380	-14.92	down	0.00029965	0.022663565	chr16
MSTRG.33589.2	11203	-15.12	down	5.06E-06	0.001290824	chr6
MSTRG.43000.11	2397	-15.16	down	1.03E-08	7.55815E-06	chrX
MSTRG.15769.2	12765	-15.28	down	1.42E-06	0.000489261	chr16
MSTRG.41808.2	824	-15.47	down	4.41E-05	0.004723303	chr9

Table S2. The detailed information of primer sequences

Primer names	sequences(5'to3')
MSTRG.11938.2-F	GCTCAAGGCAGAACAGGCTATGG
MSTRG.11938.2-R	TTGTTGGTTGCTTGCTTGCTTTGG
MSTRG.1811.2-F	ACAGACAACAGCAAGCAGGAAGAC
MSTRG.1811.2-R	CAGAGGGGAGAGGGAGTGAAACC
MSTRG.1615.1-F	GTCCTCTGACCACCCACAAATGC
MSTRG.1615.1-R	TGTCCCTCCATCTGTCCATCCATC
MSTRG.33589.2-F	GAGGGTGGGTTCTGAATGTAATGGG
MSTRG.33589.2-R	TTGTGCTTGCTGACCTATTGCTACC
MSTRG.43000.11-F	GCGTTAGAGACAGACCCAGATTTCC
MSTRG.43000.11-R	CACAGGTCAGAGTGCTCCATTGC
MSTRG.15769.2-F	CACTTCTCTGGTCTCTGGCACTTTC
MSTRG.15769.2-R	ATGGACTTGGAGAGGGAACAGGAG
m-miR-383-3p-F	AACAATCCACAGCACTGCCT
m-miR-383-3p-R	CAGTGCAGGGTCCGAGGT
m-miR-383-3p-RT	GTCGTATCCAGTGCAGGGTCCGAGGTATTCGCA CTGGATACGACTCTGAC
m-miR-199b-5p-F	CGCGCCCAGTGTTCAGACTAC
m-miR-199b-5p-R	AGTGCAGGGTCCGAGGTATT
m-miR-199b-5p-RT	GTCGTATCCAGTGCAGGGTCCGAGGTATTCGCA CTGGATACGACGAACAG
m-miR-152-3p-F	AGGTGTTCTCAGTGCATGACA
m-miR-152-3p-R	CAGTGCAGGGTCCGAGGT

m-miR-152-3p-RT	GTCGTATCCAGTGCAGGGTCCGAGGTATTCGCA CTGGATACGACCCAAGT
m-miR-667-3p-F	ATTGCCTGACACCTGCCAC
m-miR-667-3p-R	CAGTGCAGGGTCCGAGGT
m-miR-667-3p-RT	GTCGTATCCAGTGCAGGGTCCGAGGTATTCGCA CTGGATACGACCTTGGG
m-miR-3084-3p-F	AAGCACTTCTGCCAGTCTCC
m-miR-3084-3p-R	CAGTGCAGGGTCCGAGGT
m-miR-3084-3p-RT	GTCGTATCCAGTGCAGGGTCCGAGGTATTCGCA CTGGATACGACTGTCTG
m-miR-216b-5p-F	GCGAAATCTCTGCAGGCAA
m-miR-216b-5p-R	AGTGCAGGGTCCGAGGTATT
m-miR-216b-5p-RT	GTCGTATCCAGTGCAGGGTCCGAGGTATTCGCA CTGGATACGACTCACAT
m-ENSMUST024639-F	ACAAGAGAAGGAAGCCCAACACAAG
m-ENSMUST024639-R	GTGGCGAAGGTGGCAGATTAGC
m-ENSMUST027020-F	CACACGGAACACGGCATCTACTG
m-ENSMUST027020-R	ATGGGCAGGGTGGTGGGTAATC
m-ENSMUST102888-F	CACACCACTTCCTTCCACCACTTC
m-ENSMUST102888-R	AGCATTCCATACAAGAGCCAAACCC
m-ENSMUST017548-F	GCTGAGGCTCGCAGGTCTAA
m-ENSMUST017548-R	GCCAGGCCGCTATGTACAGA
m-ENSMUST120737-F	GATAACCACTTCCGATGAGCCAGAC
m-ENSMUST120737-R	AGCAGCGAGCACAGCAAAGTAC
m-ENSMUST046542-F	GAAGCCGCTGTGTCGTGTGTAG
m-ENSMUST046542-R	GCTCTTCCCTCTGGCATGTTTGG

Table S3. The detailed information of the top five up-regulated and down-regulated mRNAs

mRNA name	length	Gene id	log_2FC	regulation	*P*-value	FDR	chromosome
ENSMUST00000106933	5191	Dnajc6	18.58	up	2.59E-07	0.000118285	chr4
ENSMUST00000179365	2464	Snx10	17.87	up	4.51E-13	3.14738E-09	chr6
ENSMUST00000033163	1823	Mettl9	17.44	up	2.31E-06	0.000720954	chr7
ENSMUST00000069994	2258	St3gal5	17.14	up	2.70E-06	0.000827866	chr6
ENSMUST00000031862	1889	Cbx3	16.80	up	5.38E-06	0.001361614	chr6
ENSMUST00000098190	5008	Rragd	-17.22	down	8.51E-07	0.000322074	chr4
ENSMUST00000022816	3372	Sub1	-17.37	down	4.16E-06	0.001134237	chr15
ENSMUST00000178474	2565	Actr3	-17.62	down	1.12E-14	1.70314E-10	chr1
ENSMUST00000082424	2013	Selenop	-17.63	down	4.47E-06	0.001176372	chr15
ENSMUST00000152117	2572	Ttc3	-17.94	down	4.33E-06	0.001163543	chr16

Table S4. The detailed information of differentially expressed miRNAs

miRNA name	miRNA seq	Regulation	*P*-value	Expression level	homology in human
mmu-let-7f-5p	TGAGGTAGTAGATTGTATAGTT	up	0.004	high	hsa-let-7f-5p: 1-22
mmu-miR-667-3p	TGACACCTGCCACCCAGCCCAAG	down	0.005	middle	No matches
mmu-miR-28a-3p	CACTAGATTGTGAGCTGCTGGA	up	0.005	middle	hsa-miR-28-3p: 1-22
mmu-miR-3084-3p	TTCTGCCAGTCTCCTTCAGACA	down	0.008	middle	No matches
mmu-miR-152-3p	TCAGTGCATGACAGAACTTGG	up	0.009	middle	hsa-miR-152-3p: 1-21
mmu-miR-3572-3p	TTACACTTGTCCTTCTTTCCCCAGT	down	0.013	middle	No matches
PC-3p-25230	TCCAACTTGGCCCCTTCCAGT	down	0.020	low	No matches
mmu-miR-216b-5p	AAATCTCTGCAGGCAAATGTGA	down	0.026	middle	hsa-miR-216b-5p: 1-22
mmu-miR-369-5p	AGATCGACCGTGTTATATTCGC	up	0.028	middle	hsa-miR-369-5p: 1-22
mmu-miR-551b-3p	GCGACCCATACTTGGTTTCAGT	up	0.029	middle	hsa-miR-551b-3p: 1-21
hsa-mir-1260b-p3	TCCCACCGCTGCCACCATT	down	0.030	low	hsa-mir-1260b-p3
hsa-mir-1260b-p5	TCCCACCGCTGCCACCATT	down	0.030	low	hsa-mir-1260b-p5
mmu-let-7g-5p	TGAGGTAGTAGTTTGTACAGTT	up	0.030	high	hsa-let-7g-5p: 1-22
mmu-miR-133a-5p	AGCTGGTAAAATGGAACCAAAT	up	0.033	low	hsa-miR-133a-5p: 1-22
cpo-miR-16a-5p	TAGCAGCACGTAAATATTGGA	up	0.034	middle	hsa-miR-16-5p: 1-20
mmu-miR-199b-3p	ACAGTAGTCTGCACATTGGTT	up	0.035	middle	hsa-miR-199a-3p: 1-21
mmu-miR-30e-3p	CTTTCAGTCGGATGTTTACAGC	up	0.036	middle	hsa-miR-30e-3p: 1-22
mmu-miR-98-5p	TGAGGTAGTAAGTTGTATTGTT	up	0.036	middle	hsa-miR-98-5p: 1-22
mmu-let-7i-5p	TGAGGTAGTAGTTTGTGCTGTT	up	0.039	high	hsa-let-7i-5p: 1-22
PC-3p-12229	ACAGTCAAAGCCCCTGCCTCGT	down	0.039	middle	No matches
mmu-miR-139-5p	TCTACAGTGCACGTGTCTCCAGT	down	0.040	high	hsa-miR-139-5p: 1-23
mmu-miR-199b-5p	CCCAGTGTTCAGACTACCTGTTC	up	0.043	middle	hsa-miR-199a-5p: 1-23

mmu-miR-199a-5p	CCCAGTGTTCAGACTACCTGTTC	up	0.043	middle	hsa-miR-199a-5p: 1-23
PC-3p-33965	TCTGCTCTGCTGGGCCGCGCTT	down	0.043	middle	No matches
mmu-miR-222-3p	AGCTACATCTGGCTACTGGGTCT	down	0.045	high	hsa-miR-222-3p: 1-21
mmu-miR-383-3p	CCACAGCACTGCCTGGTCAGA	up	0.046	middle	hsa-miR-383-3p: 1-19
mmu-miR-540-3p	AGGTCAGAGGTCGATCCTGGGC	down	0.047	middle	No matches
mmu-miR-708-5p	AAGGAGCTTACAATCTAGCTGGG	up	0.047	middle	hsa-miR-708-5p: 1-23
mmu-miR-126a-3p	TCGTACCGTGAGTAATAATGC	up	0.048	high	hsa-miR-126-3p: 1-21
